# Evaluating the protective effects of mouthguards with neutralizing agents against chlorinated water-induced enamel erosion

**DOI:** 10.3389/froh.2024.1469228

**Published:** 2024-09-05

**Authors:** Kemporn Kitsahawong, Waranuch Pitiphat, Pawin Thongpaiboon, Sasitorn Thongpaiboon, Sutthiphon Saengsuwannarot

**Affiliations:** ^1^ Department of Preventive Dentistry, Faculty of Dentistry, Khon Kaen University, Khon Kaen, Thailand; ^2^ Dental Division, Phra Yuen Hospital, Khon Kaen, Thailand; ^3^ Dental Division, Chok Chai Hospital, Nakhonratchasima, Thailand

**Keywords:** dental erosion, mouthguards, toothpaste, swimming, tooth demineralization

## Abstract

**Introduction:**

Dental erosion is a common problem among swimmers. This study evaluated the effects of mouthguard use with or without neutralizing agents, compared to no mouthguard use, on the microhardness of dental enamel after a swimming simulation.

**Methods:**

Ninety-six human premolars were randomly allocated into six groups of 16 each: Group A (no mouthguard), Group B (mouthguard only), Group C (mouthguard with fluoride toothpaste), Group D (mouthguard with fluoride-free toothpaste), Group E (mouthguard with CPP-ACP), and Group F (mouthguard with arginine-fluoride toothpaste). Enamel slabs were fixed in a wax model (Typodont Articulator) and used to fabricate mouthguards for all groups except Group A. Each specimen underwent cyclic immersion: 2 h in acidic chlorinated water (pH 3.1) followed by 22 h in artificial saliva, for 28 days, to simulate swimming exposure. The change in enamel surface hardness was measured using a Vickers hardness tester. All groups underwent microhardness testing, scanning electron microscopy, and polarized light microscopy.

**Results:**

The enamel hardness significantly decreased in all groups after the swimming simulation (paired *t*-test, *P*-values < 0.001), except for Group F, which used a mouthguard with arginine-fluoride toothpaste [mean reduction: 17.9 kg/mm^2^, 95% confidence interval (CI): −1.9, 37.7, *P*-value = 0.07]. Group A, without a mouthguard, exhibited the highest reduction in enamel surface hardness (mean: 190.6 kg/mm^2^; 95%CI: 177.4, 203.9), significantly differing from all other groups with mouthguards (*P*-values < 0.001). However, no statistically significant differences were observed in enamel hardness reduction among the mouthguard groups. SEM micrographs illustrated rough, irregular erosion patterns and several deep porous areas on enamel surfaces of Group A. In contrast, all mouthguard groups showed enamel surfaces similar to sound tooth surfaces. A polarized light microscopic study revealed the deepest dark areas on the enamel surface of Group A.

**Conclusions:**

Mouthguards significantly reduced enamel microhardness loss compared to no mouthguard use. While no significant differences were found among mouthguard groups with or without neutralizing agents, those lined with arginine-fluoride toothpaste showed the least enamel loss, suggesting its potential protective effect. Within the limitations of this *in vitro* study, further clinical trials are needed to validate these results.

## 1 Introduction

Dental erosion, the irreversible loss of tooth structure caused by acids and/or chelating agents without microorganism involvement, is a common problem among swimmers (1–3). While global estimates of its prevalence vary widely, ranging from 0% to 100%, a rough estimate based on available data suggests a mean prevalence of 30%−50% in deciduous teeth and 20%−45% in permanent teeth (4).

In Thailand, studies have shown a particularly high prevalence of dental erosion among swimmers, reaching approximately 90%–100% (5–7). These studies, along with others conducted across the globe (1, 2, 8–18), suggest a strong link between prolonged exposure to swimming pool water and increased risk of dental erosion. Therefore, competitive swimmers are at a high risk due to their prolonged exposure to chlorinated pool water with low pH, especially when inadequate monitoring and buffering practices fail to address the issue (19).

Dental erosion is a cumulative irreversible process. Without proper management approaches, it may continue to progress. Severe cases of dental erosions can lead to tooth structure loss and hypersensitivity, typically involving several teeth or the entire arch, necessitating costly and challenging treatments (2, 3). Erosions are important in the long-term management of oral health. Therefore, exploring methods to prevent dental erosion is crucial for developing practical, innovative management and prevention strategies to slow progression and minimize the need for complex treatments (20).

An *in vitro* study demonstrated that mouthguards can mitigate the severity of dental erosion, although they do not offer absolute protection (21). Previous research has also shown that close-fitting mouthguards are effective in preventing tooth erosion in competitive swimmers (22, 23). A case report documented successful management of dental erosion and associated hypersensitivity in a patient who used a soft mouthguard during swimming and followed up with fluoride mouthwash (24). Furthermore, several protective agents, including fluoride, casein phosphopeptide-amorphous calcium phosphate (CPP-ACP), and arginine-containing toothpaste, have been shown to promote tooth remineralization and provide relief from tooth hypersensitivity (25–28). CPP-ACP might exert protective effects on dental erosion by suppressing demineralization and enhancing remineralization (29, 30). A previous study found that it increased the hardness of eroded enamel (31). A systematic review of *in vitro* studies revealed that CPP-ACP showed promise in reducing tooth wear and increasing enamel microhardness, while different fluoride toothpastes had no significant effect on dental erosion. Due to methodological limitations inherent in *in vitro* studies, the authors concluded that these results should be considered with caution, and further studies are needed to establish the best strategy for dental erosion treatment (32). This highlights a gap in research, as numerous studies focus on remineralizing agents alone, while few explore the impact of combining them with mouthguards for dental erosion management.

Dental erosion can be difficult to measure quantitatively. Reduction in enamel hardness is often assumed to represent mineral loss due to enamel softening. Surfaces microhardness testing is a method that complements indirect measures of mineral changes (33). Therefore, this *in vitro* study investigated the effects of mouthguard use with or without various neutralizing agents, compared to no mouthguard use, on dental enamel surface microhardness after simulated swimming in chlorinated water with a low pH.

## 2 Materials and methods

This *in vitro* experimental study was approved by the Center for Ethics in Human Research, Khon Kaen University, Thailand (Protocol Number HE602001), as exempt research.

### 2.1 Specimen preparation

Ninety-six sound human premolars, extracted for orthodontic reasons, were used in this study. After extraction, all teeth were stored in 0.1% thymol solution for no more than 2 months before use. Each tooth was cut to prepare a crown segment using a diamond saw (Mecatome T180, Brié-et-Angonnes, France). Seventy-two specimens were prepared for surface microhardness testing. Crown segments were embedded in self-curing clear acrylic resin to fit into the microhardness tester. Twelve specimens were prepared for surface enamel examination using a scanning electron microscope (SEM) (HITACHI S-3000N Scanning Electron microscope, Osaka, Japan), and the remaining 12 specimens were prepared for polarized light microscopy analysis (NIKON eclipse lv100 Polarized light microscope, Kanagawa, Japan).

The specimens were randomly allocated into 6 groups: Group A (no mouthguard), Group B (mouthguard only), Group C (mouthguard with fluoride toothpaste), Group D (mouthguard with fluoride-free toothpaste, Group E (mouthguard with CPP-ACP), Group F (mouthguard with arginine-and-fluoride-containing toothpaste).

### 2.2 Microhardness measurements

Microhardness measurements of the enamel surfaces were performed using a Vickers indenter attached to a microhardness tester (Microhardness tester FM-800, Kawasaki, Japan). The pyramid shaped indentation load was 500 g (34) with 15 s dwell time. At each time point, three indentations were made per specimen: one in the central region of the dental enamel and the other two spaced 120 μm apart from each other. Each indentation was measured three times, and the mean value was calculated from triplicate measurements. Microhardness measurements were performed at two time points: baseline and after swimming simulation.

### 2.3 Mouthguard preparation

**Figure 1:**
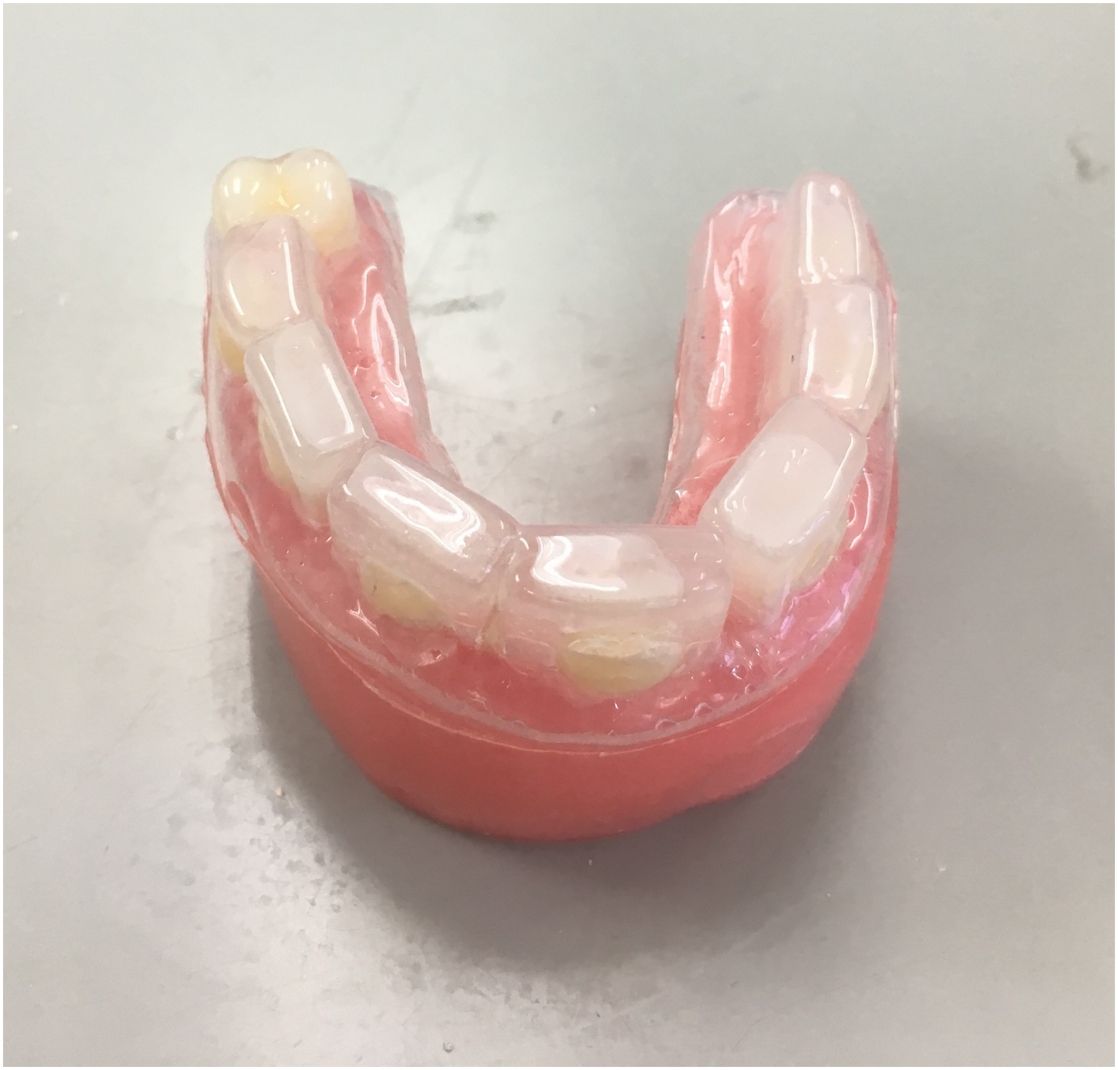
Enamel slabs fixed in a wax model and covered with a mouthguard.

The enamel slabs were fixed in a wax model (Typodont Articulator 709-0007, Ormco Corp., Glendora, CA, USA). Alginate impressions were then taken of the wax model with the enamel slabs in all groups except Group A to create mouthguards from ethyl vinyl acetate (Philips Zoom EVA Tray Material, Discus Dental, Culver City, CA, USA), as shown in Figure 1. In Group B, the enamel slabs were treated only with mouthguards. In Group C, the mouthguard was lined inside with 2.5 ml of 1,000 ppm fluoride toothpaste [Colgate fluoride toothpaste, Colgate-Palmolive (Thailand) Ltd., Chonburi, Thailand]. In Group D, the mouthguard was lined inside with 2.5 ml of fluoride-free toothpaste (Pureen kids toothpaste, Amlion Toothpaste Mfg. Sdn. Selangor, Malaysia). In Group E, the mouthguard was lined inside with 2.5 ml of CPP-ACP (GC Tooth Mousse, GC America INC., USA), and in Group F, the mouthguard was lined inside with 2.5 ml of toothpaste containing 8% arginine and 1,450 ppm fluoride [Colgate® Sensitive Pro-Relief™ Repair & Prevent, Colgate-Palmolive (Thailand) Ltd., Chonburi, Thailand].

### 2.4 Swimming simulation

All groups underwent swimming simulation by immersion in chlorinated water (containing 90% Trichloroisocyanuric acid) adjusted to a pH of 3.1 for 2 h daily over a period of 4 weeks. After exposure to the chlorinated water, the mouthguards were removed, and the specimens were cleaned with double-deionized water (DDW) before being immersed in artificial saliva for 22 h. The swimming simulation was processed in a thermostatically controlled water bath at 37°C (15 cycles/min). Once the swimming simulation was completed, the specimens were rinsed and stored in DDW at room temperature.

### 2.5 SEM examination

Specimens were dehydrated in a desiccator cabinet with an analog hygrometer unit (Nortman) for 24 h. The samples were then mounted onto stubs using double carbon conductivity tape. A thin layer of gold coating was applied onto the samples using an automated sputter coater (Emitech sputter coater models: K500X, UK) for 2 min, and they were then scanned using a SEM (HITACHI S-3000N, Japan).

### 2.6 Polarized light microscopy analysis

After the experimental period, the samples were sectioned into 300 μm thickness using a diamond saw (Mecatome T180, Brié-et-Angonnes, France). The sections were then hand-ground using a graded series of abrasive grinding pads (P800, P1000, P1200, P2500, P4000) on a Polishing Machine (Buehler ECOMET 3, USA) until the thickness reached 150–200 μm. The specimens were then washed with DDW in an Ultrasonic Cleaner (Ultrasonic steri-cleaner UC-150, Taiwan) to remove the smear layer and contaminants from the surface. Preliminary characterization was then performed using polarized light microscopy analysis (NIKON eclipse lv100POL, Japan) and imaging software (Tarosoft®, Thailand).

### 2.7 Statistical analysis

The microhardness of surface enamel, measured in Vickers hardness number (VHN), before and after swimming simulation was compared within each group using a paired *t*–test. The change in VHN before and after immersion was compared among groups using one-way ANOVA followed by the *post hoc* Bonferroni test at a 5% significance level.

## 3 Results

Table 1 presents the microhardness of surface enamel before and after swimming simulation. The VHN values significantly decreased after immersion in all groups (paired *t*-test, *P*-value < 0.001), except in Group F (*P*-value = 0.07). The highest reduction was observed in the group without a mouthguard (Group A, mean difference = 190.6), while the lowest reduction was observed in the group with arginine-fluoride toothpaste in the mouthguard (Group F, mean difference = 17.9). When compared among groups, the microhardness reduction of Group A without a mouthguard was significantly greater than that of all the mouthguard groups (*P*-value < 0.001). However, there were no statistically significant differences among the different mouthguard groups (all pairwise comparisons, *P*-values > 0.05). While the greatest difference in microhardness reduction was seen between Group E and F, this difference was not statistically significant (*P*-value = 0.06).

**Table 1:** Microhardness of surface enamel before and after swimming simulation (unit: vickers hardness number).

Swimming simulation	Control	Experimental group					Between group *P*-value (ANOVA)
	Group A	Group B	Group C	Group D	Group E	Group F	
Before simulation, mean (SD)	351.4 (13.3)	347.9 (13.5)	356.3 (12.1)	352.3 (16.2)	345.4 (12.6)	345.0 (18.5)	0.38
After simulation, mean (SD)	160.7 (25.2)	307.6 (24.9)	321.0 (22.2)	306.7 (30.5)	295.3 (39.5)	327.1 (25.5)	<0.001
Mean reduction (95% CI)	190.6 (177.4, 203.9)	40.5* (27.7, 52.7)	35.0* (22.8, 47.4)	45.6* (31.2, 60.1)	50.1* (26.2, 73.9)	17.9* (−1.9, 37.7)	<0.001
Within group *P*-value (paired *t*–test)	<0.001	<0.001	<0.001	<0.001	<0.001	0.07	

Control group: Group A (no mouthguard); Experimental group: Group B (mouthguard only), Group C (mouthguard with 1,000 ppm fluoride toothpaste), Group D (mouthguard with fluoride-free toothpaste), Group E (mouthguard with CPP-ACP), Group F (mouthguard with arginine-and-fluoride-containing toothpaste).

SD, standard deviation; CI, confidence interval.

* Significantly different from the control group (Group A) at *P*-value < 0.001.

**Figure 2:**
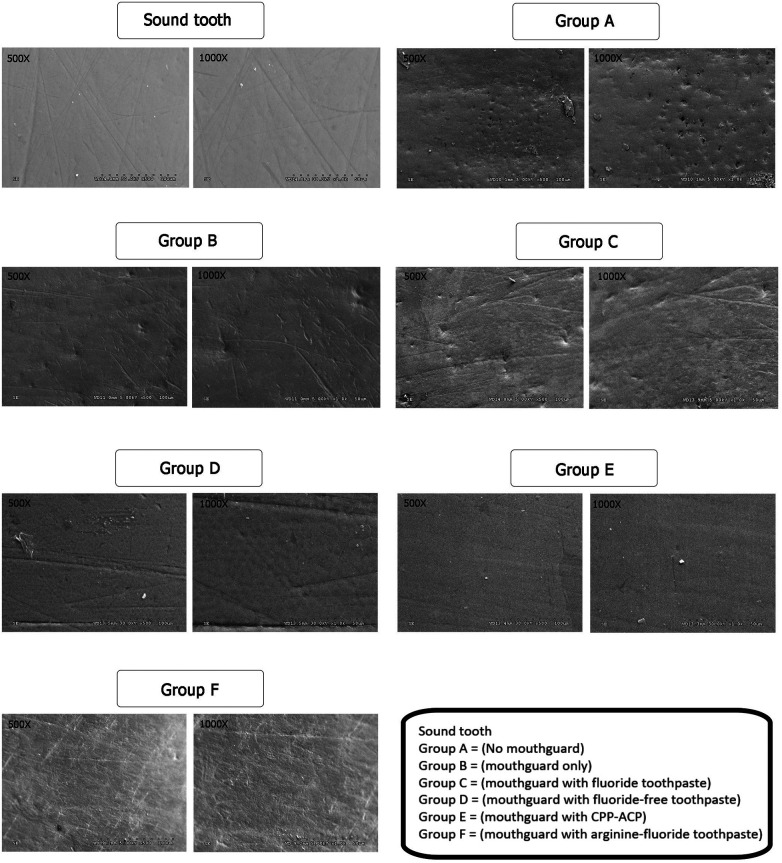
SEM images of the enamel slabs after swimming simulation using ×500 and ×1,000 magnifications.

SEM observation of sound enamel surfaces (no immersion) revealed various structures, including grooves, *perikymata*-type formation, and small depressions and ditches (Figure 2). However, the enamel surface of Group A, without mouthguard, showed irregular patterns of erosion and several deep porous areas. In contrast, specimens from the other groups with mouthguard (with/without neutralizing agents) had enamel surface structures similar to the sound enamel. Nevertheless, the enamel surface of Group B (mouthguard only) and Group C (mouthguard with fluoride toothpaste) showed some small shallow porous areas.

**Figure 3:**
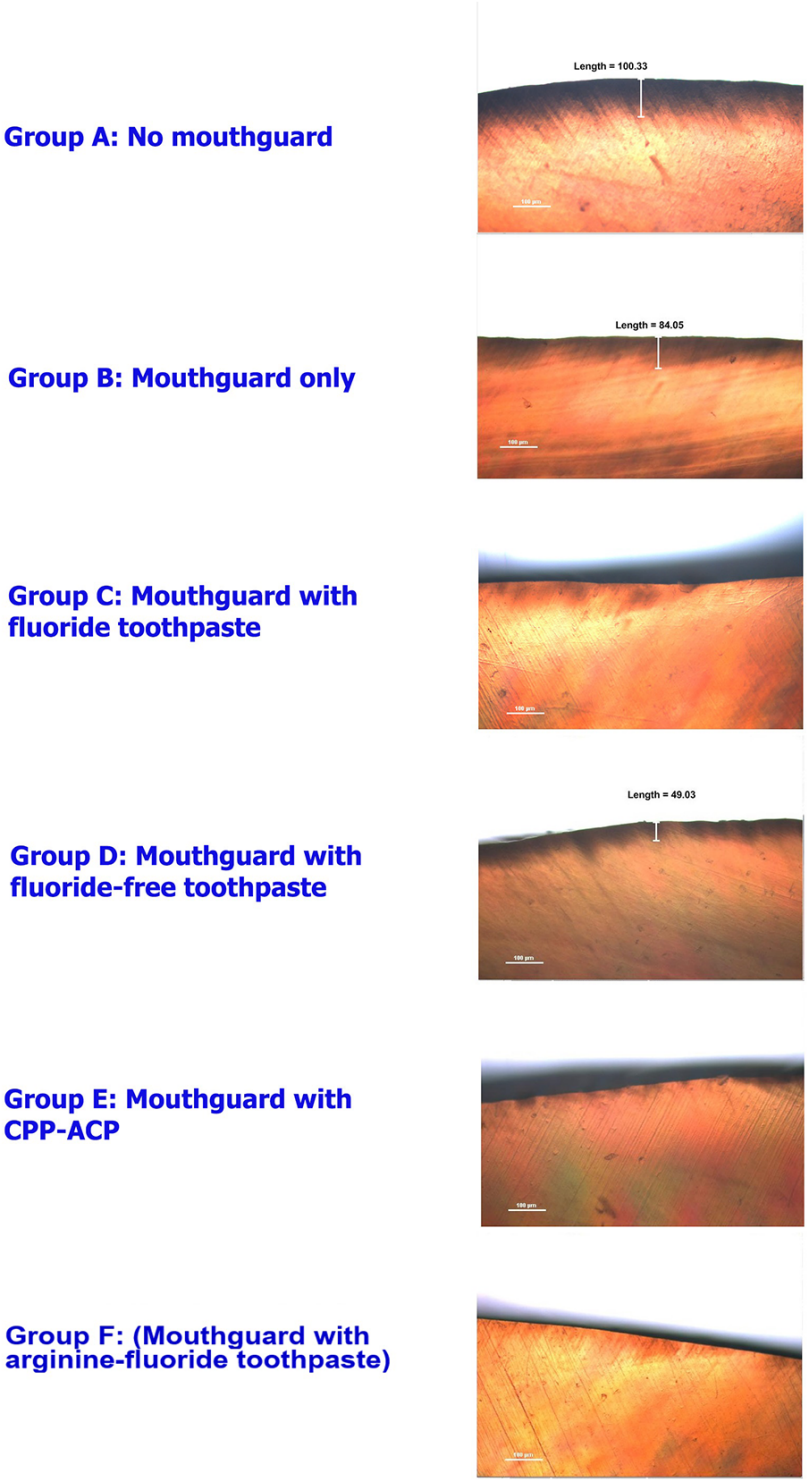
Polarized light microscope images of the enamel slabs after swimming simulation in ×10 magnifications.

Demineralization of the enamel was examined using polarized light microscope. The representative images of specimens after swimming simulation are shown in Figure 3. Polarized light microscope analysis showed the highest depth of demineralized surface enamel in the enamel slabs without mouthguard, followed by the mouthguard only group. Specimens from the mouthguard groups with neutralizing agents had only slightly demineralized areas.

## 4 Discussion

The results of this *in vitro* study indicate that dental enamel exposed to acidic chlorinated water for a prolonged duration led to dental erosion, and the use of a mouthguard could provide a protection against erosion. In addition, the use of commercially available neutralizing agents with the mouthguard may increase its ability to prevent demineralization of the enamel.

Epidemiological data have shown a high prevalence of dental erosion among swimmers (2, 11). A recent study reported that the prevalence of dental erosion was significantly higher among competitive swimmers (60%) compared to non-swimmers (25.6%) (35). Chlorine is commonly used as an antimicrobial agent in swimming pool water, but its acidic pH can contribute to tooth erosion in frequent swimmers. Dental erosion is likely to occur when the water is acidic, *i.e.,* with a pH lower than 4.5, resulting in enamel marked by undersaturation of both hydroxyapatite (at a critical pH of 5.5) and fluorapatite (at a critical pH of 4.5) structures (16). A previous study reported that products with a pH below 4 caused tooth erosion, while those with a pH above 4 did not (36). Although health authorities recommend maintaining a pH of 7.2–8.0 in swimming pools to safeguard swimmers’ health (2, 8), the lower pH of swimming pool water could occur due to inadequate buffering or insufficient monitoring (8).

A previous study in Virginia, USA, reported that a pool water sample had a pH of 2.7 (2). Similarly, a study in Egypt demonstrated that pool water showed a continuous reduction in pH over time, reaching a highly acidic level of pH 3.2 (35). A survey of water samples from two chlorinated swimming pools in South Thailand showed that the lowest pH value was 2.91 (9). Another study collecting data from swimming pools in North Thailand (7) observed the lowest mean water pH value of 3.26. These values were below the recommended standard pH range for swimming pool water (pH = 7.2–8.0). Furthermore, in a previous evaluation of swimming pools in Khon Kaen province, Northeast Thailand, we found the lowest pH to be 3 (21). These data support the relevance of using a pH of 3.1 to study its effects in the current study, as it falls within the observed range of acidic conditions that can lead to dental erosion. Nonetheless, we acknowledge that while pH 3.1 reflects a realistic scenario, erosion can occur across a broader spectrum of acidic conditions. Therefore, the results should be interpreted with caution, particularly when generalizing to environments with pH levels closer to the recommended range for swimming pools.

An incidence of rapid and excessive dental erosion in a competitive swimmer due to gas-chlorinated pool water within 27 days has been reported (14). Additionally, swimming in low-pH water for two hours a day for four weeks has been suggested to result in dental erosion (9). Therefore, this study used immersion in chlorinated water at pH 3.1 for two hours, followed by immersion in artificial saliva for 22 h daily for four weeks to simulate swimming conditions. The reduction in microhardness test results of this *in vitro* study confirmed that immersion in low-pH water for two hours a day demineralizes tooth tissue, resulting in dental erosion. Similarly, a cross-sectional study in Szczecin, Poland, reported that competitive swimmers who trained for more than 19 h per week in closely monitored gas-chlorinated swimming pools had a greater risk of dental erosion than recreational swimmers who swam for no more than two hours per week (1).

Slow cumulative effects of dental erosion can be very difficult to detect, primarily due to the lack of stable and reproducible reference points on tooth surfaces. In this study, we used microhardness measurements to evaluate dental erosion because the reduction in microhardness is likely a result of mineral loss from the tooth surfaces (37). Various methods are employed for microhardness measurements. For instance, the indentation load ranges from 25 to 500 g, with dwell times varying between 15 and 20 s (31, 38–40). Additionally, a previous study by Meredith et al. (1996) reported that loads of 0.98 N (99.93 g) for dentine and 4.9 N (499.66 g) for enamel produced indentations suitable for measuring the short and long indentation diagonals and caused minimal tooth surface damage (34). The indentation load of 500 g with a 15-s dwell time used in our experimental study falls within the commonly accepted range for enamel microhardness testing. We also used SEM to examine the alterations of surface enamel structure and polarized light microscopy to examine the depth of demineralization. Human premolars extracted for orthodontic purposes were used as study specimens, as they are typically sound teeth suitable for microhardness testing.

Treatment of dental erosion can be complex and costly; thus, prevention is the preferred option. Regular fluoride application is recommended to prevent dental erosion in swimmers (41). Both monovalent and polyvalent fluoride compounds, such as tin fluoride toothpaste, have been shown to protect against enamel and dentin erosive tooth wear (42). In addition, a case report demonstrated satisfactory results in patients suffering from dental erosion when they wore mouthguards during swimming (24). Furthermore, an *in vitro* study reported that mouthguard use reduced the severity of dental erosion by protecting teeth from acids in chlorinated water. However, it is important to note that a mouthguard cannot provide complete protection, as some acidic water can still penetrate the mouthguard (21). The use of a mouthguard with a neutralizing agent may promote tooth remineralization and alleviate tooth hypersensitivity. This, in turn, enhances tooth protection against dental erosion.

The present study focused on neutralizing agents that are commercially available in Thailand, aiming to offer practical solutions that swimmers can easily incorporate into their routines. Group C used a fluoride toothpaste with 1,000 ppm fluoride, the most commonly available concentration at the time of the study. In contrast, Group F used an arginine-fluoride toothpaste with 1,450 ppm fluoride, as a 1,000 ppm fluoride toothpaste containing arginine was not available in Thailand. This approach underscores the real-world applicability of the findings. However, testing specific ingredients such as fluoride, CPP-ACP, and arginine at controlled concentrations could provide more detailed insights into their individual effectiveness. Future research could explore these specific ingredients to further refine protective strategies against enamel erosion in chlorinated swimming environment.

The results of this study showed that enamel hardness significantly decreased in all groups after the swimming simulation, except for the group using a mouthguard with arginine-fluoride toothpaste. The group without a mouthguard exhibited the greatest decrease in hardness, which was significantly different from all the mouthguard groups. There were no statistically significant differences observed among the various mouthguard groups. Thus, while lining a mouthguard with neutralizing agents tended to result in less reduction in enamel microhardness than using a mouthguard alone, the differences were not statistically significant.

The SEM analyses did not indicate any distinct differences in enamel surface alteration among enamel slabs with mouthguards compared to sound enamel surfaces. In contrast, enamel slabs in the “no mouthguard” group exhibited irregular patterns of erosion and generalized deep porosities on their enamel surfaces. While previous studies have frequently shown honeycomb-etched patterns of erosive demineralization (21, 41, 43) the present study did not find such honeycomb-etched patterns. One possible explanation for the observed differences is the variation in the simulation process. Previous studies immersed the tooth in acid-chlorinated water only, without subsequent remineralization by saliva (21, 41). Another study incubated the samples in a remineralizing solution consisting of artificial saliva and stimulated human saliva for 4 h after an erosive challenge (43). In the present study, we immersed the samples in acidic water for two hours followed by immersion in artificial saliva for 22 h daily. This longer remineralization period may have led to the obliteration of etched patterns on the enamel surface by the action of saliva. Saliva possesses various properties, including buffering, neutralizing erosive agents, and forming the acquired pellicle to protect the tooth surface against acid-induced demineralization (8, 44).

Polarized light microscopy was used for the qualitative assessment of dental mineralization by demonstrating demineralized areas of the lesion as a radiolucent surface layer (45). In the present study, polarized light microscopic analysis revealed that the greatest depth of demineralization on the surface enamel was observed in the group without mouthguards. High depths of demineralized areas were also present in the group with mouthguard alone. In contrast, all the mouthguard groups with neutralizing agents exhibited slightly shallower demineralized areas, with the lowest depth of demineralized area being found in a mouthguard with arginine-fluoride toothpaste group. This finding is consistent with the results of enamel hardness test especially in the mouthguard with arginine-fluoride toothpaste group, which showed the least decrease in hardness. These results suggested that neutralizing agents may promote remineralization. While the polarized light microscopic analysis provided valuable insights into the surface characteristics of the enamel, this technique cannot quantify the degree of dental erosion in the study since the sample size was relatively small. Further studies with larger sample sizes would allow for quantitative statistical analysis of the data, providing more robust results.

The present study has several other limitations. Dental erosion can be challenging to measure quantitatively, and changes in mineral density on tooth surfaces were assumed to represent mineral loss due to surface softening (37). The microhardness measurement used to evaluate dental erosion is an indirect method and may not substitute for direct quantitative measurement. Additionally, a recent study reported that intraoral scanners can be effectively used to monitor the progression of dental wear (46). Future studies may consider incorporating intraoral scanners, as they have been shown to be accurate in assessing dental wear progression. Future well-designed studies could also employ techniques such as x-ray diffraction analysis (XRD), Fourier-transform infrared spectroscopy (FTIR), energy-dispersive x-ray spectroscopy (EDS), and Raman spectroscopy to provide a more comprehensive chemical characterization.

Moreover, the laboratory setting limited the ability to replicate real-mouth characteristics and the dynamic nature of swimming environments. Although swimming conditions were simulated, this cannot fully capture the complexity of real-life situations, such as the intermittent emergence of teeth from the water, wave creation, or the muscular stretching associated with different swimming strokes. These factors could potentially influence the extent of enamel erosion observed. Future studies with improved standardization could involve distinct experiments designed to analyze the effects of various swimming strokes, incorporating mechanical simulators that better replicate the dynamic movements and environmental factors present in actual swimming conditions. Additionally, longer immersion times in chlorinated water may be necessary to more accurately reflect the practice duration of competitive swimmers.

Finally, being an *in vitro* study, the demineralization and remineralization processes may not entirely reflect the *in vivo* situations. Dental erosion from chlorinated water depends not only on the water's erosive potential but also on several biological factors, including tooth structure, saliva, and individual oral conditions. Additionally, wearing a mouthguard during swimming may protect the teeth from mechanical forces such as muscular scratching and exposure to water waves, so further clinical studies are recommended.

## 5 Conclusions

Within the limitations of the study, the following conclusions were drawn:

1. Exposure to acidic water without using a mouthguard resulted in a significant decrease in enamel microhardness compared to all mouthguard conditions (with or without a neutralizing agent.
2. Lining the mouthguard with a commercially-available neutralizing agent may further reduce demineralization, but the difference compared to using a mouthguard alone was not statistically significant.
3. Lining the mouthguard with arginine-and-fluoride-containing toothpaste resulted in the least reduction in microhardness, with no statistically significant difference observed between pre- and post-exposure microhardness values. This suggests its potential effectiveness in protecting enamel during swimming.

Further clinical trials supplemented with *in vitro* studies are needed to authenticate the results for future clinical applications.

## Data Availability

The raw data supporting the conclusions of this article will be made available by the authors, without undue reservation.
